# Tick (Acari: Ixodidae) infestation of cuscuses from Maluku Province, Indonesia

**DOI:** 10.14202/vetworld.2021.1465-1471

**Published:** 2021-06-08

**Authors:** Prasetyarti Utami, Bambang Heru Budianto, Ana Sahara

**Affiliations:** 1 Faculty of Biology, Universitas Jenderal Soedirman, Purwokerto, Central Java, Indonesia; 2 Program of Biology, Faculty of Science and Technology, Universitas Terbuka, Jakarta, Indonesia; 3 Department of Parasitology, Faculty of Veterinary Medicine, Universitas Gadjah Mada, Yogyakarta, Indonesia

**Keywords:** Cuscus, *Ixodes*, scanning electron microscope

## Abstract

**Background and Aim:**

Cuscuses are one of the endemic Indonesian marsupials, which needs to be protected and revived in terms of the numbers and range of species. Ectoparasites of ticks (Ixodidae) are one potential obstacle to cuscus conservation. Tick infestation can cause blood loss in the host, even being a predisposing factor for infection with pathogenic organisms. This study aimed to determine the prevalence, infestation intensity, and species of ticks present on cuscuses in Maluku Province, Indonesia.

**Materials and Methods:**

Ticks were collected from cuscuses origin of the four regions in Maluku Province, namely the island of Ambon, Seram Island, Romang Island, and Wetar Island. Cuscuses were captured at night, with ticks being collected from them from the head to the tip of the tail. The tick samples obtained from the cuscuses were preserved, identified, and counted. Cuscuses were released back into their habitat after collecting the ticks. The obtained ticks were observed using an Olympus BX51 microscope with an Olympus DP12 digital camera and prepared for examination under a scanning electron microscope (SEM). Infestation rate, infestation intensity, and morphology of the species were described.

**Results:**

The cuscuses were found to be infested with *Ixodes cordifer* ticks. Cuscuses in Maluku Province had a low tick infestation rate. The range of infestation prevalence of island origin cuscuses in Maluku was between 14.28% and 16.67%. Simultaneously, *I. cordifer* infestation level was mildly infested based on the intensity of thick infestation ranged from 1 to 1.2 ticks per cuscus. From observation of the tick surface structure under SEM, sexual dimorphism and various specific characteristics of the ticks were identified.

**Conclusion:**

The low infestation rate of *I. cordifer* ticks in cuscus was influenced by the up and down movement of the conscious activity in the tree, which allowed minimal contact with the ticks. The infestation prevalence rates on each island studied were similar. Such similarities of infestation are related to the similarity of cuscus species among Ambon, Lakor, Seram, and Romang islands, which are all included in the Phalangeridae family, and their similar habitats, behaviors, climatic conditions, and geographical areas.

## Introduction

Cuscus is a marsupial of the Phalangeridae family with a relatively wide distribution, which is especially prevalent in East Indonesia and Papua New Guinea. The distribution of cuscus in Indonesia is restricted to the east of the country, in Maluku, Sulawesi, Papua, and Timor [1]. Several species of cuscus are critically endangered and headed for extinction (vulnerable), such as woodlark cuscus (*Phalanger lullulae*), Gebe cuscus (*Phalanger alexander*), blue-eyed spotted cuscus (*Spilocuscus wilsoni*), Telefomin cuscus (*Phalanger matanim*), black-spotted cuscus (*Spilocuscus rufoniger*), Talaud bear cuscus (*Ailurops melanotis*), Waigeo cuscus (*Spilocuscus papuensis*), bear cuscus (*Ailurops ursinus*), and blue-eyed cuscus (*Phalanger matabiru*). There are six cuscus genera globally, namely, Ailurops, Phalanger, Spilocuscus, Strigocuscus, Wyulda, and Trichosurus, with 28 species [2]. Four genera of the Phalangeridae are distributed in Indonesia, with several species endemic to Papua, the Maluku Islands, and the Sulawesi Islands There are approximately 24 species of cuscus in Indonesia, but they are becoming increasingly rare. Flannery [3] reported that *Phalanger orientalis* was scattered from Timor, Wetar, the Maluku Islands of Misool, Batanta, Salawati, and New Guinea to the Bismarck islands on the eastern side of Papua New Guinea.

*Spilocuscus* spp. have been reported to be scattered in the southern part of the Maluku Islands (Buru, Seram, Banda, and Ambon), Aru Islands, Kei Islands, Misool, Yapen, and the mainland of New Guinea (Papua), as well as to inhabit a small part of Cape York Peninsula in North Australia [4].

In Indonesia, cuscus has been protected since 1979 by the prohibition on the capture of such animals, as set out in the Order Minister of Agriculture No. 247/KPTS/UM4/1979, Wild Animal Hunting Regulation (PPBL) No. 226/1931, Law No. 5/1990 concerning the conservation of living natural resources and their ecosystems, and Law No. 7/1999 concerning the preservation of plant and animal species. According to CITES data (Convention on International Trade in Endangered Species of Wild Fauna and Flora), several Indonesian marsupial species are endangered, so they are included in Appendix II, while IUCN data (International Union for Conservation of Nature and Natural Resources) classify them as endangered species (IUCN, 2008). Kunda *et al*. [5,6] stated that the decline in the cuscus population has been significantly influenced by hunting by humans, while genetic factors have also contributed. Illegal hunting is the basis for trading in its illegal meat, as it is consumed as a protein source and to meet meat needs during church ceremonies [7,8].

Pathologically, all members of marsupials are susceptible to pathogenic infections by bacteria, viruses, or parasites [9]. Ticks can cause health problems such as anemia, dermatitis, and secondary infections [10]. The ectoparasites found in almost all groups of marsupials are hard ticks belonging to the genus *Ixodes*. Ticks of the genus *Ixodes* Latreille, 1795, are common parasites of reptiles, birds, and mammals worldwide. The tick species *Ixodes cordifer* has been found in cuscuses in Indonesia [11,12], but the infestation rate and intensity of tick infestation have not been reported. There is also a need to describe the surface ultrastructural morphology of the ticks because no study has been carried out on ticks obtained from cuscuses in Indonesia.

This study aimed to determine the prevalence, infestation intensity, and species of ticks present on cuscuses in Maluku Province, Indonesia. There is also a need to compare detailed morphological features of the ticks with the descriptions that have been reported previously.

## Materials and Methods

### Ethical approval

This study is complementary to the ethical requirements of laboratory animals and has been approved by the Ethics Committee team of Laboratorium Penelitian dan Pengujian Terpadu Universitas Gadjah Mada (LPPT UGM).

### Study period and location

**Figure-1:**
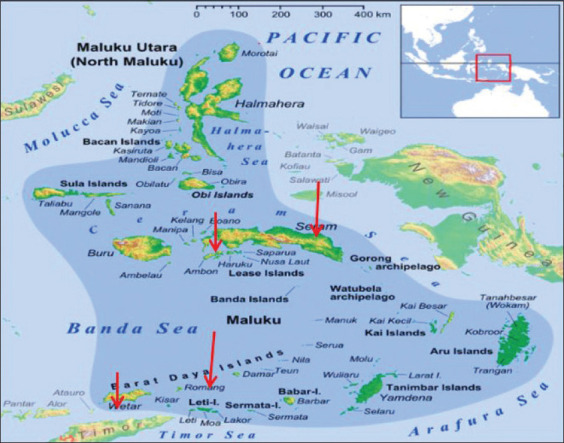
Locations of tick collection on cuscus on Wetar, Romang, Ambon, and Seram Islands (Source: https://id.wikipedia.org/wiki/Kepulauan_Maluku).

This research was conducted from August 2017 to July 2020. Collection of ticks on cuscus was carried out in Maluku Province namely Ambon Island, Seram Island, Romang Island, and Wetar Island (Figure-1). Samples were processed at Indonesian Institute of Sciences, Cibinong, Indonesia; Laboratorium Penelitian dan Penujuan Terpadu, Universitas Gadjah Mada (LPPT UGM); and Laboratory of Parasitology, Faculty of Veterinary Medicine, Universitas Gadjah Mada, Yogyakarta, Indonesia.

### Tick collection

**Figure-2:**
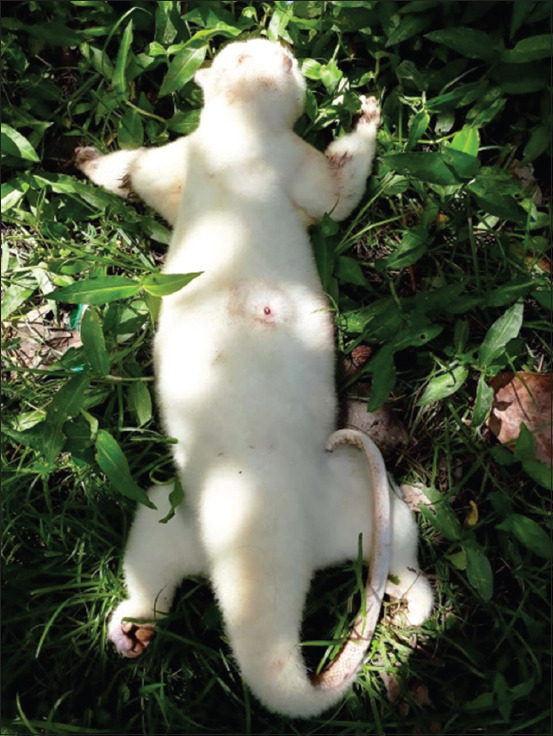
Cuscus caught and anesthetized.

Cuscuses were captured using a method involving imitation of their sounds to lure them out of the undergrowth. This is a well-recognized approach locally. When a cuscus jumped from one branch to another and reached the point where the sounds originated, it was captured with a gunny sack and immediately injected with ketamine and xylazine by veterinary personnel. In an unconscious state, each captured cuscus was carefully examined for ticks (Figure-2). Subsequently, the cuscus was allowed to return to consciousness and injected with 0.5 ml of Vitamin B complex, after which it could return to its habitat. The ticks obtained from the cuscuses were prepared on slides and examined by scanning electron microscopy (SEM).

### Morphological identification

The morphological characteristics of the ticks were observed with an Olympus BX51 microscope and an Olympus DP12 digital camera. Ultrastructural observation was performed by SEM with a JEOL JSM-651OLA. The observed features included overall appearance, and anterior and posterior parts. SEM preparations were performed through Integrated Research and Testing Laboratory Gadjah Mada University procedures. Identification was performed based on morphological features using an identification key [13-16].

### Statistical analysis

The structure of the ticks was analyzed descriptively according to existing references. The level of tick infestation was categorized according to the intensity of the ticks. When between 1 and 25 ticks were found on one cuscus, this was categorized as mild infestation, between 26 and 50 as moderate infestation, and 51 or more as severe infestation [17]. The prevalence of tick infestation and its intensity was calculated in accordance with a previous report [18].

The determined prevalence of tick-infested cuscuses is presented as the proportion of cuscuses infected with tick relative to the total number of cuscus samples. The intensity of tick infestation was determined based on comparison between the average number of individual ectoparasite species and the number of infested cuscuses.

## Results

**Figure-3:**
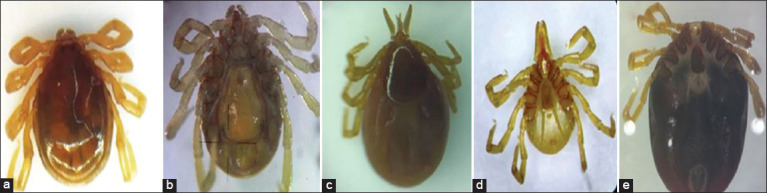
Morphology of *Ixodes cordifer*. (a) Dorsal (male); (b) ventral (male); (c) dorsal (female); (d) ventral (female); (e) ventral engorged (female). Magnification 20x.

The ticks collected from cuscuses from Ambon, Wetar, Romang, and Seram were morphologically examined microscopically and identified as members of the genus *Ixodes* through the presence of a distinct anal groove anterior of the anus. The *Ixodes* ticks were in the subgenus *Sternalixodes* [16,19]. One of the characteristics of this subgenus is the presence of a sternal plate on the ventral part of the body. The morphology of male and female *I. cordifer* is shown in Figure-3. The genital aperture was situated at the level between coxae I and II in males and at the mid-fourth intercostal space in females. Male *I. cordifer* was first described by Neumann [13]. Male *I. cordifer* from Maluku has an elongated oval body, with a glossy scutum covering the entire dorsum. The male body is brownish-yellow and dark. All males have legs of the same brown color. The palps are relatively short and wide, but narrower than the basis capituli.

**Figure-4:**
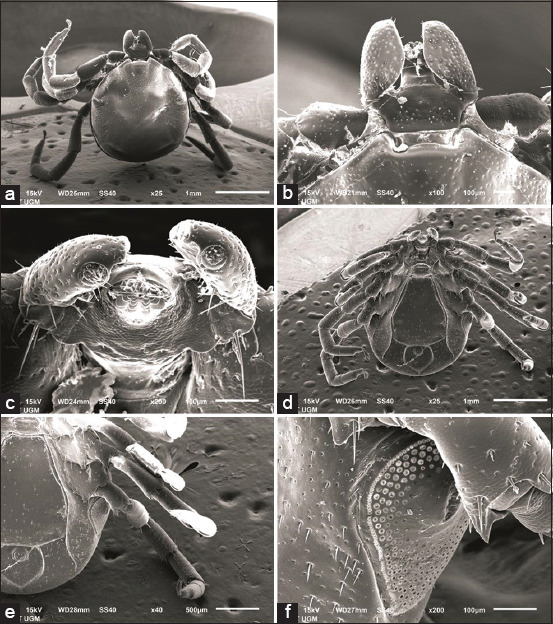
Scanning electron microscopy of *Ixodes cordifer* male. (a) Dorsal; (b) Gnathostoma; (c) dentition; (d) ventral; (e) coxae I-IV; (f) spiracle. Scale bars: (a) 1 mm; (b and c) 100 μm; (d) 1 mm; (e) 500 μm; (f) 100 μm.

The males were found to be moderate in size, with a body length of 2.6-2.95 mm and width of 1.861-1.931 mm, and a capitulum length of 0.34-0.42 mm and width of 0.32-0.4 mm (Table-1). Palpal article 1 is transverse, while palpal articles 2-3 are fused and then narrow. The male ticks’ hypostome is characterized by two short and blunt teeth (Figure-4). The scutum has a linear lateral carina; the punctuation size is small and sparse distribution on conscutum. The male ticks have two spurs on coxae 1-3, with the internal spurs being inconspicuous while the spurs on coxa 4 are single and pointed. There are short lateral carinae with tapered scapulae. The base of the capituli is pentagonal in shape, the dorsal part does not have cornua and does not have auricula on the ventral side. *I. cordifer* has scattered, fine, and short hair. The median plate is broad and quadrangular in shape. The pregenital plate is located on coxa 2 and has an adanal plate. The anal groove joins toward the posterior. The anal plate is heart shaped and tapers in males.

**Table-1:** Morphometry of male and female *Ixodes cordifer* (mm).

Location	BL	LG	LI	WI	LS	WS	LDCB	WDCB
Ambon								
F	6.394	1.098	5.296	3.308	1.822	1.867	0.425	0.547
M	2.95	0.347	2.603	1.861	2.55	1.861	0.2	0.328
F	4.52	1.05	3.50	2.933	1.462	1.721	0.4	0.43
F	3.99	0.94	3.05	2.35	1.420	1.690	0.38	0.42
Wetar								
F	3.115	0.954	2.161	2.02	1.877	1.910	0.41	0.472
F	10.5	1.086	9.414	8.82	1.650	1.848	0.53	0.57
F	3.15	0.92	2.23	1.91	1.850	1.930	0.39	0.42
F	8.80	1.15	7.65	6.90	1.890	1.960	0.5	0.54
F	7.75	1.080	6.67	5.70	1.852	1.882	0.52	0.56
Seram								
M	2.625	0.421	2.204	1.931	2.125	1.931	0.30	0.407
F	7.63	1.080	6.55	5.550	1.820	1.925	0.384	0.415
F	8.71	1.12	7.59	6.772	1.874	1.954	0.47	0.53
F	7.56	1.08	6.48	5.82	1.877	1.901	0.47	0.49
F	6.85	1.07	5.78	3.50	1.855	1.870	0.44	0.564
F	7.44	1.08	6.36	5.56	1.860	1.895	0.47	0.57
Romang								
F	4.619	0.9	3.719	2.754	1.454	1.790	0.49	0.52
F	4.55	0.9	3.650	2.963	1.436	1.762	0.473	0.482
F	5.119	1.060	4.059	3.061	1.691	1.820	0.51	0.57
F	4.650	0.988	3.662	2.967	1.540	1.785	0.48	0.5
F	4.700	1.016	3.684	2.676	1.601	1.799	0.501	0.746
Average	5.78	0.97	4.91	3.97	1.78	1.86	0.08	0.09
SD	2.26 5.78±2.26	0.22 0.97±0.22	2.14 4.91±2.14	2.03 3.97±2.03	0.27 1.78±0.27	0.08 1.86±0.08	0.44 0.44±0.08	0.50 0.504±0.09

F=Female, M=Male, BL=Body length, LG=Length of *Gnathostoma*, LI=Length of idiosoma, WI=Wide of idiosoma; LS=Length of scutum, WS=Wide of scutum, LDCB=Length of dorsal capitulum base, WDCB=Wide of dorsal capitulum base

**Figure-5:**
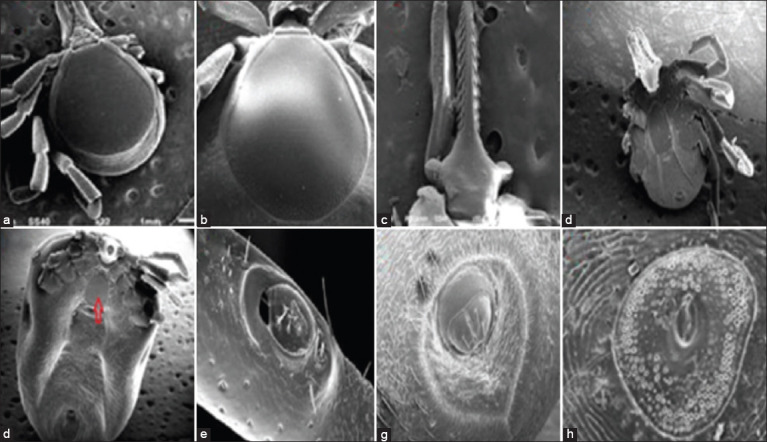
Scanning electron microscopy of *Ixodes cordifer* female from Maluku Province, Indonesia. (a) Dorsal; (b) scutum; (c) teeth arrangement of female; (d) ventral; (e) ventral of engorged; (f) Haller‘s organ; (g) anal; (h) spiraculum plate. Scale bars: (a) 1 μm; (b) 50 μm; (c) 200 μm; (d-f) 1 mm; (g-h) 100 μm. Arrow: Sternal plate.

Female *I. cordifer* is 3.15-10.5 mm in length and 1.9-8.8 mm in width (Table-1), idiosoma ovoid widest at the posterior end of the scutum. The capitulum in female ticks is longer than that in males. The hypostome of female ticks is characterized by elongated and sharp teeth lanceolate. The base of the capituli does not have a cornua and has a separate porous area. Punctuations on the scutum are dense and small at the posterior. The morphology of the tick is more clearly visible on the scanning electron microscopy figure. Female ticks have a 3:3 tooth arrangement (Figure-5). The lateral part of the carina is well developed, while the base of the capituli is pentagonal, having a slender elongated palpal with transverse palpal article 1 and fused palpal articles 2/3. Female ticks have a round scapula. The auricula in females was found at the ventral base of the capituli. This is consistent with a previous finding [16] that *I. cordifer* has auricula without cornua. The genital opening is located in coxa 4, while the sternal plate is oval. The anal groove embraces the anus anteriorly, forming an arch and it is joined at the posterior to form a point.Coxae I-IV have external single spurs with a hill-like shape, while the spiracular plate is subcircular or broadly oval. The general shape of Haller’s organ is oval to elliptical, along with an accessory pit with seven setae, not tightly clustered in the center, with two large setae and five small setae.

Cuscuses in Ambon, Wetar, Seram, and Romang had low rates of infestation with *I. cordifer*. The species of cuscus infested with the tick *I. cordifer* are *Phalanger orientalis* and *Spilocuscus maculatus*. Not all cuscuses found on the islands of Ambon, Wetar, Seram, and Romang were infested with ticks. *I. cordifer* was found on cuscuses at a low infestation rate as fewer than 25 ticks were found per animal on each island. Observations of *I. cordifer* ticks found in cuscuses from Maluku showed that the collected ticks had an average intensity of 1.06 The rates of *I. cordifer* infestation among cuscuses from islands in Maluku ranged between 14.28% and 16.67% (Table-2). Meanwhile, *I. cordifer* intensity in cuscuses from Maluku ranged from 1 to 1.2. The caught cuscuses were *P. orientalis* and *P. maculatus*, with ticks more commonly being found on the cuscus abdomen (Table-3).

**Table-2:** Prevalence and intensity of *Ixodes cordifer* on cuscus.

No.	Region	Location	Number of cuscus	Infested cuscus	Number *Ixodes cordifer* collected	Prevalence (%)	Intensity
1	Ambon	Negeri Suli, Gunung Sahulutu	25	4	4	16	1
2	Wetar	Desa Ustutun	35	5	5	14.28	1
3	Seram	Outside Manusela National Park	30	5	6	16.67	1,2
4	Romang	Desa Jerusu	35	5	5	14.28	1
			125	19	20		
Average						15.2%	1.06

**Table-3:** Region, cuscus species infested.

No.	Island	Cuscus infested *I. cordifer*	Tick species	Attached on bodies	Number of tick
1	Ambon	*P*. *orientalis* (female)	*I. cordifer* (female)	Abdomen	1
		*P*. *orientalis* (male)	*I. cordifer* (female)	Abdomen	1
		*P*. *orientalis* (male)	*I. cordifer* (female)	Leg	1
		*P*. *orientalis* (male)	*I. cordifer* (male)	Abdomen	1
2	Wetar	*P*. *orientalis* (male)	*I. cordifer* (female)	Thorax	1
		*P*. *orientalis* (male)	*I. cordifer* (female)	Abdomen	1
		*P*. *orientalis* (male)	*I. cordifer* (female)	Abdomen	1
		*P*. *orientalis* (male)	*I. cordifer* (female)	Abdomen	1
		*P*. *orientalis* (male)	*I. cordifer* (female)	Thorax	1
3	Seram	*S. maculatus* (female)	*I. cordifer* (male)	Abdomen	1
			*I. cordifer* (female)	Thorax	1
		*P*. *orientalis* (male)	*I. cordifer* (female)	Abdomen	1
		*P*. *orientalis* (male)	*I. cordifer* (female)	Abdomen	1
		*P*. *orientalis* (male)	*I. cordifer* (female)	Abdomen	1
		*S. maculatus* (male)	*I. cordifer* (female)	Thorax	1
4	Romang	*P*. *orientalis* (male)	*I. cordifer* (female)	Axilla	1
		*P*. *orientalis* (male)	*I. cordifer* (female)	Cervic	1
		*P*. *orientalis* (male)	*I. cordifer* (female)	Dorsum	1
		*P*. *orientalis* (male)	*I. cordifer* (female)	Abdomen	1
		*P*. *orientalis* (male)	*I. cordifer* (female)	Leg	1

*I. cordifer=Ixodes cordifer*, *P*. *orientalis=Phalanger orientalis*

## Discussion

In Indonesia, the genus *Ixodes* consists of only five species: *I*. *granulatus*, *I. spinicoxalis*, *I. werneri*, and *I. kopsteini* [14]. The first three species are parasitic on rats, while the last is on bats. Two females *Ixodes cordifer* were found from cuscuses on Ambon Island [20]; later, the tick was found in the bear cuscus (*Ailurops ursinus*) at Sulawesi [11].

One individual male tick of *I. cordifer* was previously identified from Sekru (New Guinea), the western part of Irian Jaya [12], and successfully described [13], but its host was unknown. It was also previously reported [15] that several tick species from Papua were successfully identified as *I. cordifer* with *Phalanger* sp. as a host. However, reports on the tick *I. cordifer* [11-13] did not include a morphological description or detailed explanation. This limited amount of information is due to the difficulty of collecting samples and the rarity of ticks on cuscuses, as well as the biological activity of this marsupial.

This study identified as many as 20 *I. cordifer*, including 2 males and 18 females. These ticks were found on cuscuses on the islands of Ambon, Water, Seram, and Romang. The identified ticks were uniform in type. They were similarly distributed on these islands in Maluku, which is unsurprising given the similar environmental conditions, with similar supplies of resources and similar interactions between *I. cordifer* and its hosts.

There is clear sexual dimorphism of this tick species in the auricula. In males, no auricula was seen on the ventral side, which is consistent with a previous description of male *I. cordifer* [13]. In contrast, female *I. cordifer* has an auricula on the ventral side, with matched character matrics of *Ixodes* belonged to the subgenus Sternalixodes is largely present auricular [16]. The morphology of the spurs of coxae I-IV also differs between males and females. The male coxae I-III have two spurs (external and internal ones), with the internal spur not being conspicuous. Coxa IV has one external spur with a tapered shape and no internal spur.

In general, cuscuses hide in trees during the day, at the height of 15 m above the ground. They hide in tree holes and branches with dense canopy to avoid predators. The natural predators of cuscuses include several reptile species (e.g. snakes), but humans are now the main predators threatening the survival of these marsupials. During the day, this species generally hides in tree holes, tree crowns, and at night this species moves foraging by jumping from tree to tree to pick up fruit and seeds, where it possibly contacts larvae of *I. cordifer*. However, the results of this study prove that contact between cuscus and *Ixodes* occurs very rarely. Few *Ixodes* were found on cuscuses and they were extremely difficult to collect. This was also influenced by the season. Based on our observations, season is a significant determinant of the success or failure of *Ixodes* sp. to reach the host’s (cuscus) body. During the dry season*, Ixodes* actually had difficulty finding cuscuses. However, *Ixodes* could be found in cuscuses during the rainy season. It is possible that, during the dry season, *Ixodes* could not withstand the heat and lacked water. Thus, it might have made some adaptations by being active on vegetation close to the ground to save energy. It was previously suggested by Estrada Pena [21] that ticks were affected by season in a manner characterized by their upward and downward movement in vegetation. Cuscus behavior was also influenced by the growth of fruit during the dry season.

It was previously reported by Viggers *et al*. [22] that the *I. trichosuri* tick found in *Trichosurus caninus* marsupial from Cambarville, Australia, had an average intensity of 1.6, while *I. tasmania* had an average intensity of 1. The intensity findings for *Ixodes* in Maluku resembled those for Australian marsupials. This shows that *Ixodes* ticks found in cuscuses and other marsupial groups are related to the hosts foraging or performing other activities at night, which determines the likelihood of contacting the tick.

The abdomen and the chest were chosen by *I. cordifer* as anatomical sites to attach themselves. The abdomen and the chest are the sites frequently in contact with trees when cuscuses look for food. Cuscuses were particularly likely to contact ticks when on trees looking for food. It was reported by Latif *et al*. [23] that predators such as rodents, birds, reptiles, ants, and pathogens such as fungi could limit the habitat of tick larvae when searching for a host. Birds, which were considered as a host of *I. cordifer* larvae, could possibly limit the ability of the tick *I. cordifer* to find cuscuses by bringing it outside of the cuscus’s geographical range.

## Conclusion

There is an external spur on the coxae of *I. cordifer* obtained from cuscuses in Maluku, where cuscuses have a low rate of *I. cordifer* infestation. This is probably due to the lack of opportunity for *I. cordifer* to encounter cuscuses and the difficulty of gathering samples of ticks on cuscuses given that they are arboreal and nocturnal animals. Infestations of *I. cordifer* ticks found in cuscuses from Maluku showed that the ticks that were collected had an average intensity of 1.06. The average prevalence of *I. cordifer*-infested cuscuses from islands in Maluku was 15.2%. The prevalence and intensity of infestation on each island examined were relatively similar. This would be due to the similar geographical and climatic conditions in Maluku**.**

## Authors’ Contributions

PU: Collected samples, analyzed the data, conducted research in the laboratory and wrote the manuscript. AS and BHB: Delivered reagents/materials/analysis result, examined the data, wrote and critically revised the manuscript. All authors read and approved the final manuscript.
